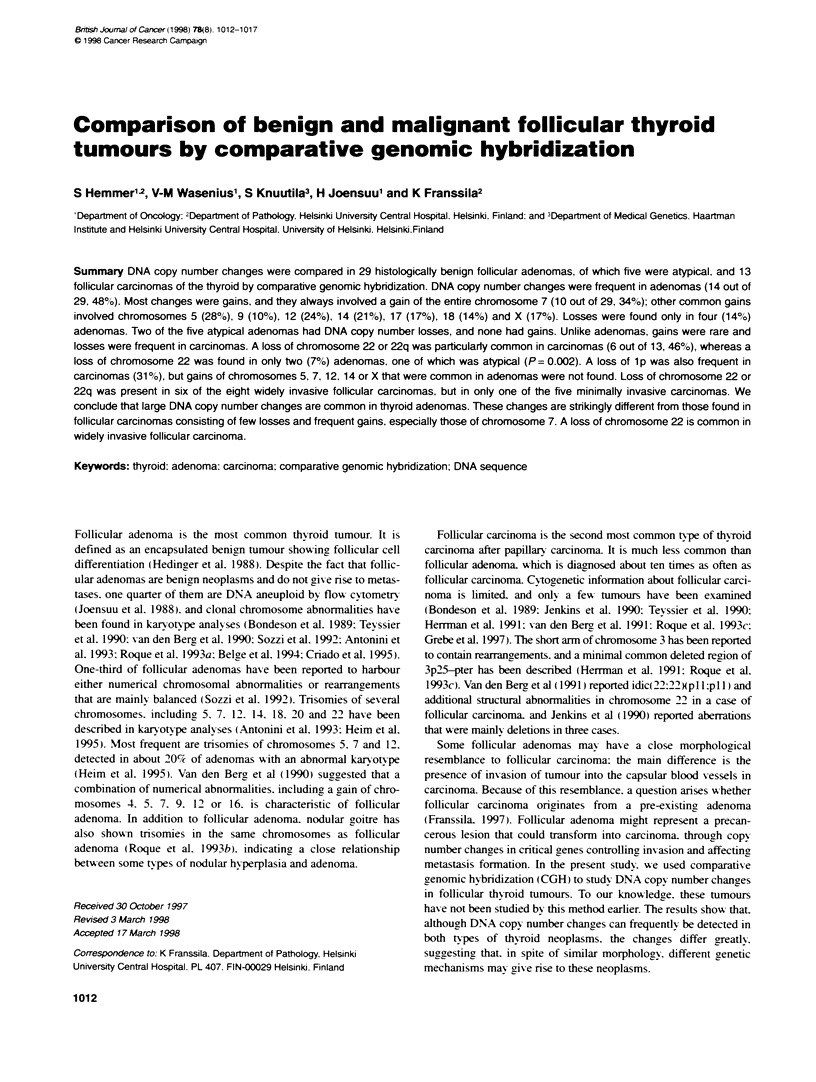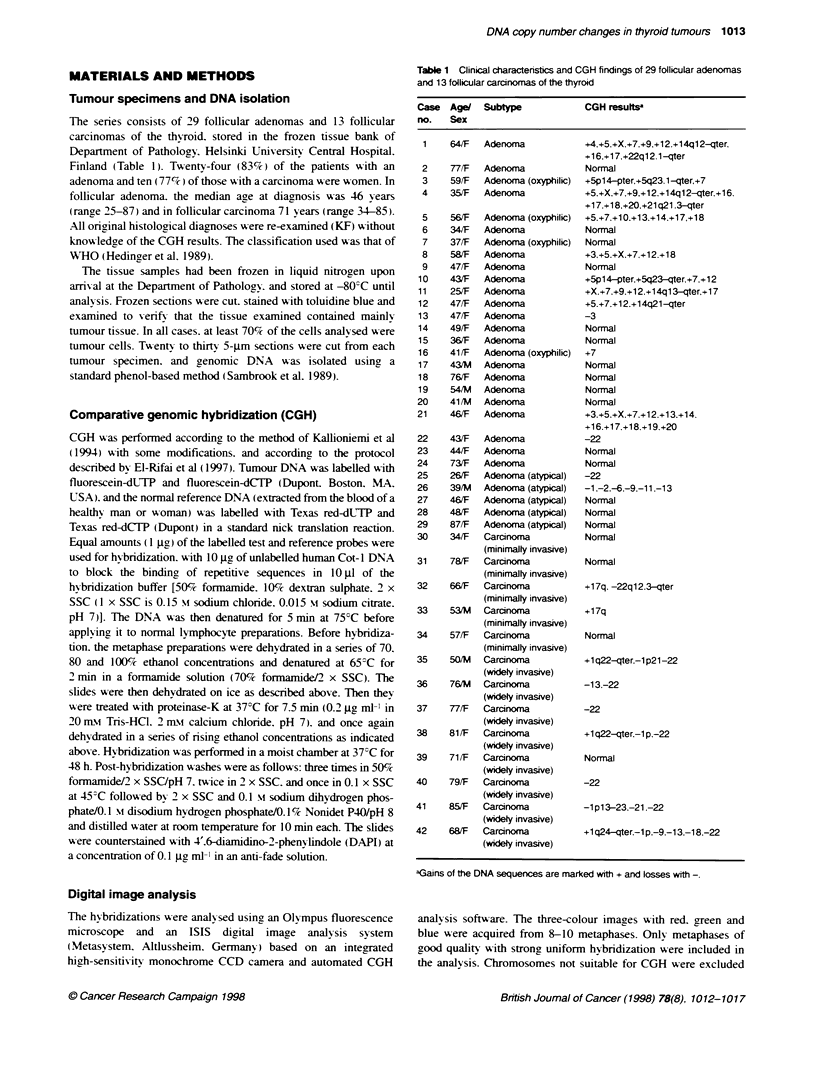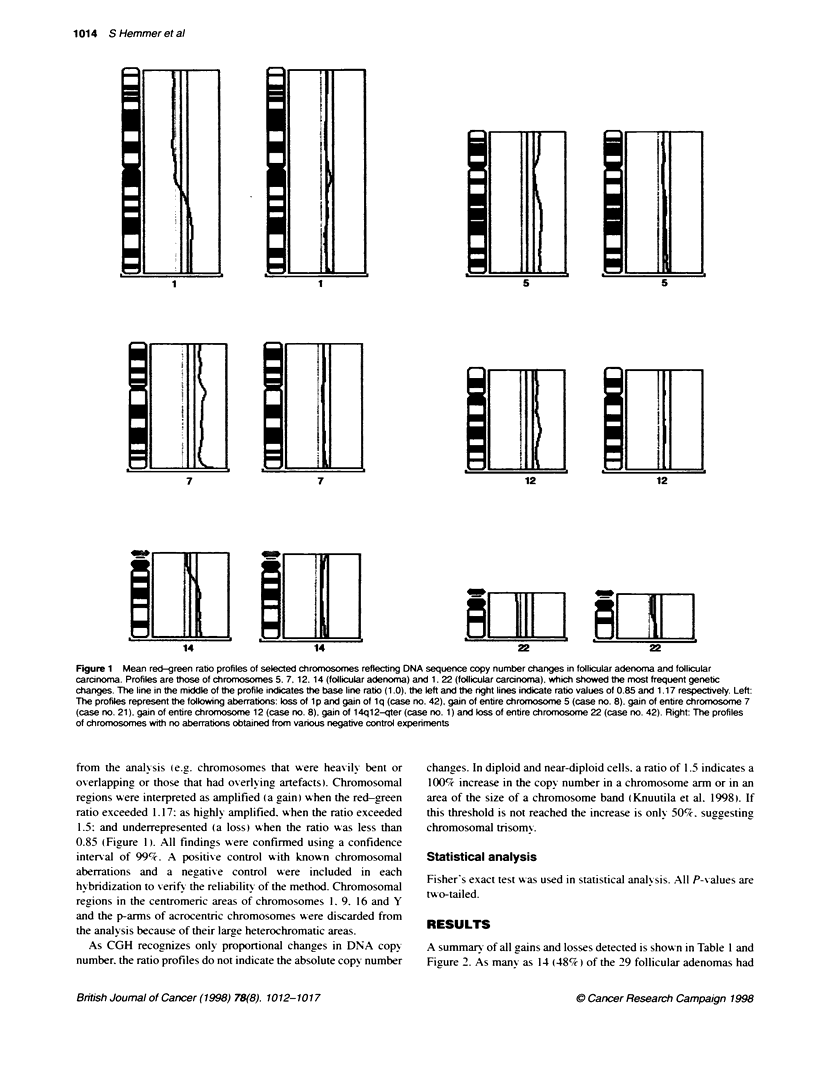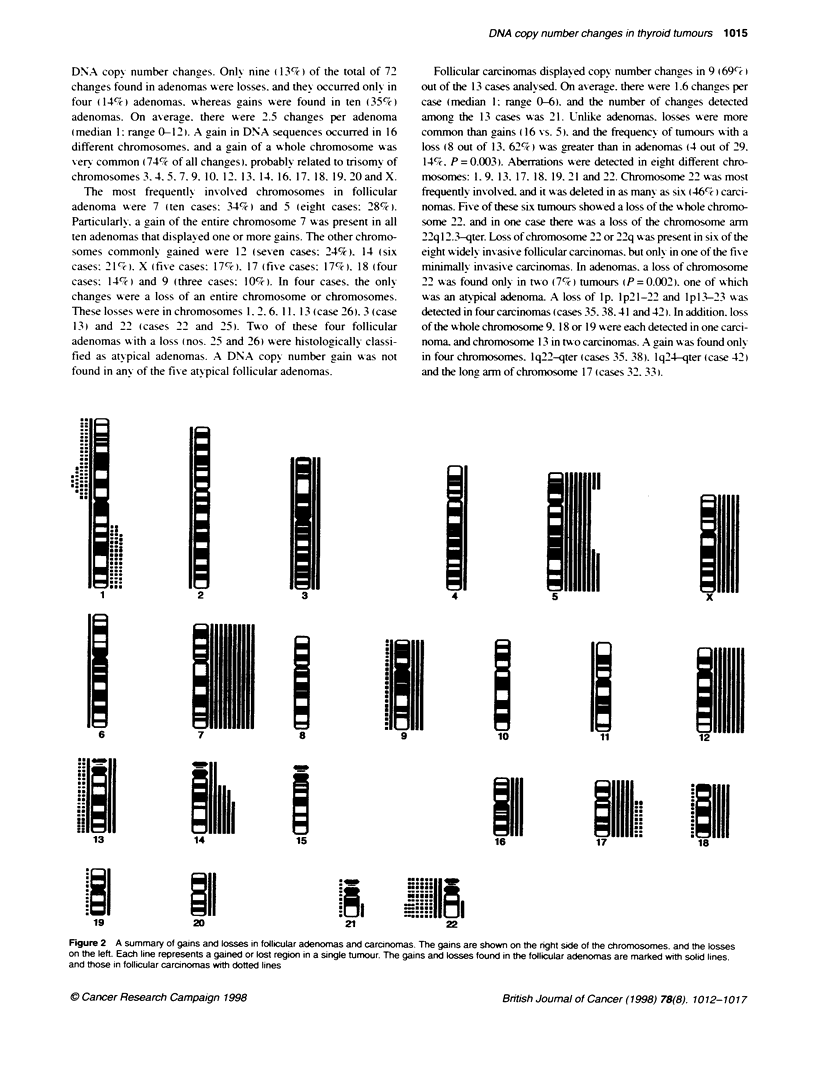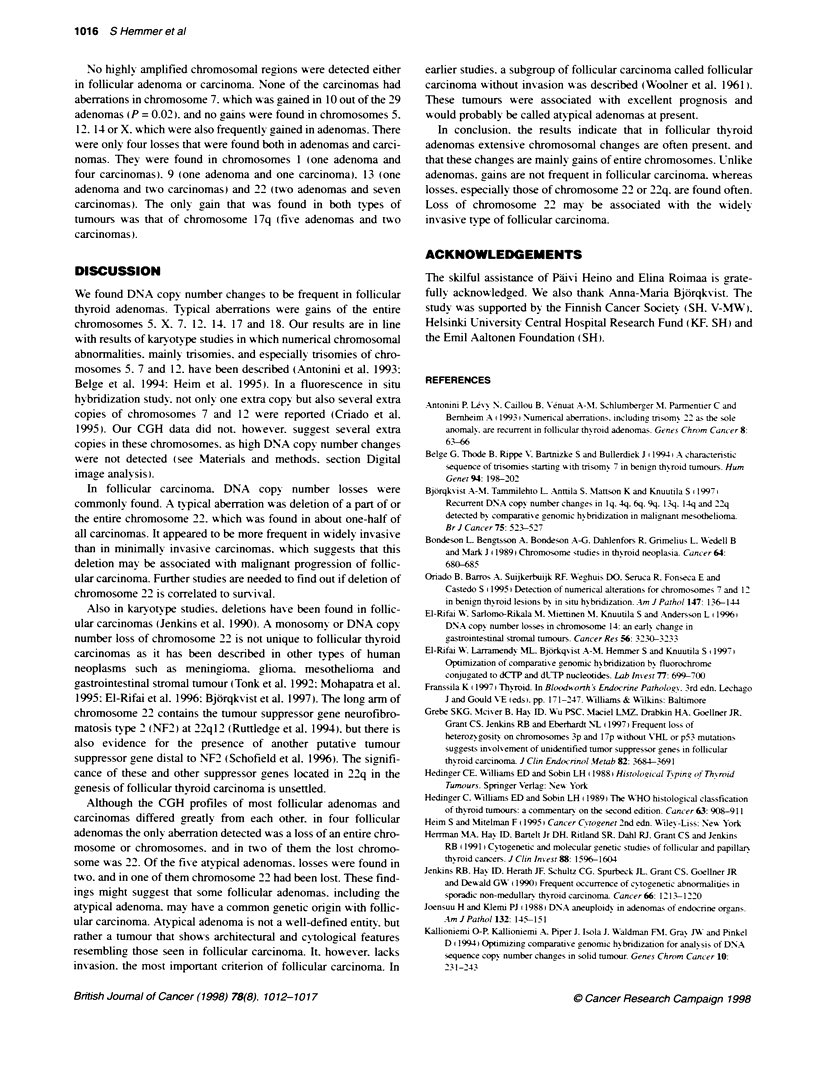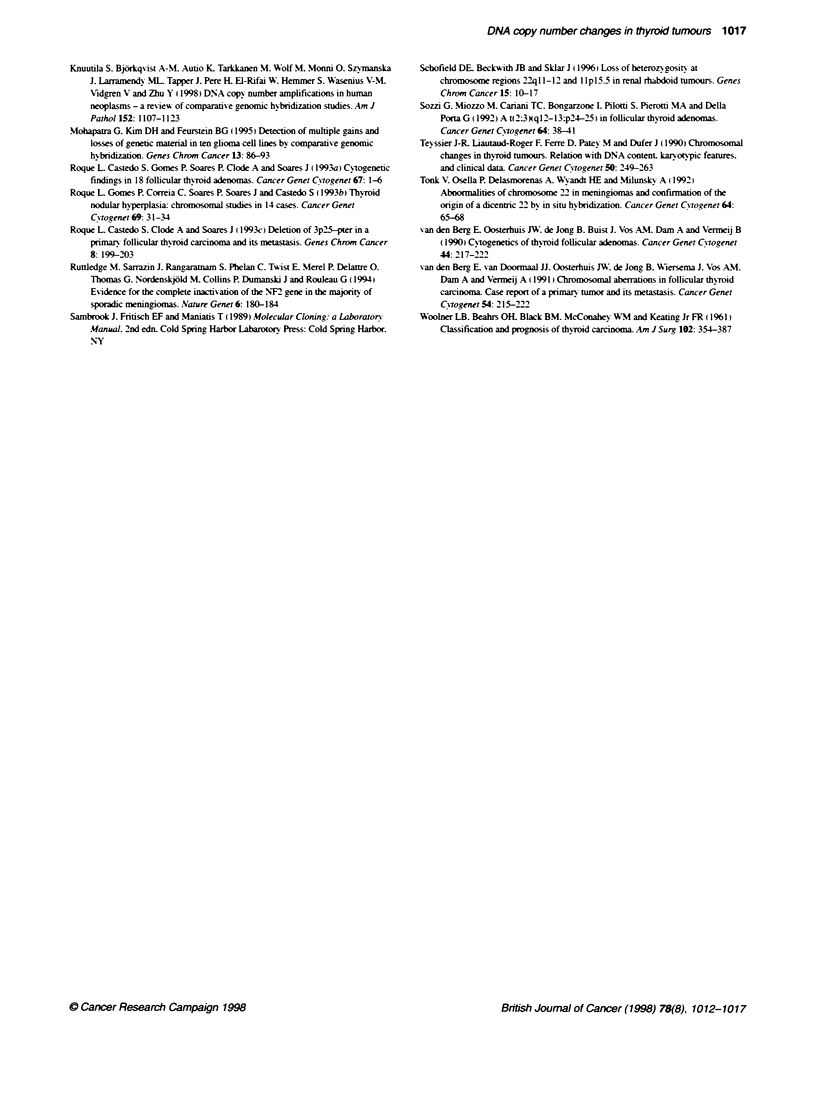# Comparison of benign and malignant follicular thyroid tumours by comparative genomic hybridization.

**DOI:** 10.1038/bjc.1998.620

**Published:** 1998-10

**Authors:** S. Hemmer, V. M. Wasenius, S. Knuutila, H. Joensuu, K. Franssila

**Affiliations:** Department of Oncology, Helsinki University Central Hospital, Finland.

## Abstract

DNA copy number changes were compared in 29 histologically benign follicular adenomas, of which five were atypical, and 13 follicular carcinomas of the thyroid by comparative genomic hybridization. DNA copy number changes were frequent in adenomas (14 out of 29, 48%). Most changes were gains, and they always involved a gain of the entire chromosome 7 (10 out of 29, 34%); other common gains involved chromosomes 5 (28%), 9 (10%), 12 (24%), 14 (21%), 17 (17%), 18 (14%) and X (17%). Losses were found only in four (14%) adenomas. Two of the five atypical adenomas had DNA copy number losses, and none had gains. Unlike adenomas, gains were rare and losses were frequent in carcinomas. A loss of chromosome 22 or 22q was particularly common in carcinomas (6 out of 13, 46%), whereas a loss of chromosome 22 was found in only two (7%) adenomas, one of which was atypical (P = 0.002). A loss of 1p was also frequent in carcinomas (31%), but gains of chromosomes 5, 7, 12, 14 or X that were common in adenomas were not found. Loss of chromosome 22 or 22q was present in six of the eight widely invasive follicular carcinomas, but in only one of the five minimally invasive carcinomas. We conclude that large DNA copy number changes are common in thyroid adenomas. These changes are strikingly different from those found in follicular carcinomas consisting of few losses and frequent gains, especially those of chromosome 7. A loss of chromosome 22 is common in widely invasive follicular carcinoma.


British Journal of Cancer (1998) 78(8), 1012–1017
© 1998 Cancer Research Campaign

### Comparison of benign and malignant follicular thyroid tumours by comparative genomic hybridization

**S Hemmer[1,2], V-M Wasenius[1], S Knuutila[3], H Joensuu[1] and K Franssila[2]**

[1]Department of Oncology; [2]Department of Pathology, Helsinki University Central Hospital, Helsinki, Finland; and [3]Department of Medical Genetics, Haartman Institute and Helsinki University Central Hospital, University of Helsinki, Helsinki, Finland

**Summary** DNA copy number changes were compared in 29 histologically benign follicular adenomas, of which five were atypical, and 13 follicular carcinomas of the thyroid by comparative genomic hybridization. DNA copy number changes were frequent in adenomas (14 out of 29, 48%). Most changes were gains, and they always involved a gain of the entire chromosome 7 (10 out of 29, 34%); other common gains involved chromosomes 5 (28%), 9 (10%), 12 (24%), 14 (21%), 17 (17%), 18 (14%) and X (17%). Losses were found only in four (14%) adenomas. Two of the five atypical adenomas had DNA copy number losses, and none had gains. Unlike adenomas, gains were rare and losses were frequent in carcinomas. A loss of chromosome 22 or 22q was particularly common in carcinomas (6 out of 13, 46%), whereas a loss of chromosome 22 was found in only two (7%) adenomas, one of which was atypical ($P = 0.002$). A loss of 1p was also frequent in carcinomas (31%), but gains of chromosomes 5, 7, 12, 14 or X that were common in adenomas were not found. Loss of chromosome 22 or 22q was present in six of the eight widely invasive follicular carcinomas, but in only one of the five minimally invasive carcinomas. We conclude that large DNA copy number changes are common in thyroid adenomas. These changes are strikingly different from those found in follicular carcinomas consisting of few losses and frequent gains, especially those of chromosome 7. A loss of chromosome 22 is common in widely invasive follicular carcinoma.

**Keywords:** thyroid; adenoma; carcinoma; comparative genomic hybridization; DNA sequence

Follicular adenoma is the most common thyroid tumour. It is defined as an encapsulated benign tumour showing follicular cell differentiation (Hedinger et al, 1988). Despite the fact that follicular adenomas are benign neoplasms and do not give rise to metastases, one quarter of them are DNA aneuploid by flow cytometry (Joensuu et al, 1988), and clonal chromosome abnormalities have been found in karyotype analyses (Bondeson et al, 1989; Teyssier et al, 1990; van den Berg et al, 1990; Sozzi et al, 1992; Antonini et al, 1993; Roque et al, 1993*a*; Belge et al, 1994; Criado et al, 1995). One-third of follicular adenomas have been reported to harbour either numerical chromosomal abnormalities or rearrangements that are mainly balanced (Sozzi et al, 1992). Trisomies of several chromosomes, including 5, 7, 12, 14, 18, 20 and 22 have been described in karyotype analyses (Antonini et al, 1993; Heim et al, 1995). Most frequent are trisomies of chromosomes 5, 7 and 12, detected in about 20% of adenomas with an abnormal karyotype (Heim et al, 1995). Van den Berg et al (1990) suggested that a combination of numerical abnormalities, including a gain of chromosomes 4, 5, 7, 9, 12 or 16, is characteristic of follicular adenoma. In addition to follicular adenoma, nodular goitre has also shown trisomies in the same chromosomes as follicular adenoma (Roque et al, 1993*b*), indicating a close relationship between some types of nodular hyperplasia and adenoma.

*Received 30 October 1997*
*Revised 3 March 1998*
*Accepted 17 March 1998*

*Correspondence to:* K Franssila, Department of Pathology, Helsinki University Central Hospital, PL 407, FIN-00029 Helsinki, Finland

Follicular carcinoma is the second most common type of thyroid carcinoma after papillary carcinoma. It is much less common than follicular adenoma, which is diagnosed about ten times as often as follicular carcinoma. Cytogenetic information about follicular carcinoma is limited, and only a few tumours have been examined (Bondeson et al, 1989; Jenkins et al, 1990; Teyssier et al, 1990; Herrman et al, 1991; van den Berg et al, 1991; Roque et al, 1993*c*; Grebe et al, 1997). The short arm of chromosome 3 has been reported to contain rearrangements, and a minimal common deleted region of 3p25–pter has been described (Herrman et al, 1991; Roque et al, 1993*c*). Van den Berg et al (1991) reported idic(22;22)(p11;p11) and additional structural abnormalities in chromosome 22 in a case of follicular carcinoma, and Jenkins et al (1990) reported aberrations that were mainly deletions in three cases.

Some follicular adenomas may have a close morphological resemblance to follicular carcinoma; the main difference is the presence of invasion of tumour into the capsular blood vessels in carcinoma. Because of this resemblance, a question arises whether follicular carcinoma originates from a pre-existing adenoma (Franssila, 1997). Follicular adenoma might represent a precancerous lesion that could transform into carcinoma, through copy number changes in critical genes controlling invasion and affecting metastasis formation. In the present study, we used comparative genomic hybridization (CGH) to study DNA copy number changes in follicular thyroid tumours. To our knowledge, these tumours have not been studied by this method earlier. The results show that, although DNA copy number changes can frequently be detected in both types of thyroid neoplasms, the changes differ greatly, suggesting that, in spite of similar morphology, different genetic mechanisms may give rise to these neoplasms.

### MATERIALS AND METHODS

### Tumour specimens and DNA isolation

The series consists of 29 follicular adenomas and 13 follicular carcinomas of the thyroid, stored in the frozen tissue bank of Department of Pathology, Helsinki University Central Hospital, Finland (Table 1). Twenty-four (83%) of the patients with an adenoma and ten (77%) of those with a carcinoma were women. In follicular adenoma, the median age at diagnosis was 46 years (range 25–87) and in follicular carcinoma 71 years (range 34–85). All original histological diagnoses were re-examined (KF) without knowledge of the CGH results. The classification used was that of WHO (Hedinger et al, 1989).

The tissue samples had been frozen in liquid nitrogen upon arrival at the Department of Pathology, and stored at –80°C until analysis. Frozen sections were cut, stained with toluidine blue and examined to verify that the tissue examined contained mainly tumour tissue. In all cases, at least 70% of the cells analysed were tumour cells. Twenty to thirty 5-μm sections were cut from each tumour specimen, and genomic DNA was isolated using a standard phenol-based method (Sambrook et al, 1989).

### Comparative genomic hybridization (CGH)

CGH was performed according to the method of Kallioniemi et al (1994) with some modifications, and according to the protocol described by El-Rifai et al (1997). Tumour DNA was labelled with fluorescein-dUTP and fluorescein-dCTP (Dupont, Boston, MA, USA), and the normal reference DNA (extracted from the blood of a healthy man or woman) was labelled with Texas red-dUTP and Texas red-dCTP (Dupont) in a standard nick translation reaction. Equal amounts (1 μg) of the labelled test and reference probes were used for hybridization, with 10 μg of unlabelled human Cot-1 DNA to block the binding of repetitive sequences in 10 μl of the hybridization buffer [50% formamide, 10% dextran sulphate, 2 × SSC (1 × SSC is 0.15 M sodium chloride, 0.015 M sodium citrate, pH 7)]. The DNA was then denatured for 5 min at 75°C before applying it to normal lymphocyte preparations. Before hybridization, the metaphase preparations were dehydrated in a series of 70, 80 and 100% ethanol concentrations and denatured at 65°C for 2 min in a formamide solution (70% formamide/2 × SSC). The slides were then dehydrated on ice as described above. Then they were treated with proteinase-K at 37°C for 7.5 min (0.2 μg $ml^{-1}$ in 20 mM Tris-HCl, 2 mM calcium chloride, pH 7), and once again dehydrated in a series of rising ethanol concentrations as indicated above. Hybridization was performed in a moist chamber at 37°C for 48 h. Post-hybridization washes were as follows: three times in 50% formamide/2 × SSC/pH 7, twice in 2 × SSC, and once in 0.1 × SSC at 45°C followed by 2 × SSC and 0.1 M sodium dihydrogen phosphate/0.1 M disodium hydrogen phosphate/0.1% Nonidet P40/pH 8 and distilled water at room temperature for 10 min each. The slides were counterstained with 4′,6-diamidino-2-phenylindole (DAPI) at a concentration of 0.1 μg $ml^{-1}$ in an anti-fade solution.

### Digital image analysis

The hybridizations were analysed using an Olympus fluorescence microscope and an ISIS digital image analysis system (Metasystem, Altlussheim, Germany) based on an integrated high-sensitivity monochrome CCD camera and automated CGH analysis software. The three-colour images with red, green and blue were acquired from 8–10 metaphases. Only metaphases of good quality with strong uniform hybridization were included in the analysis. Chromosomes not suitable for CGH were excluded

**Table 1** Clinical characteristics and CGH findings of 29 follicular adenomas and 13 follicular carcinomas of the thyroid

| Case no. | Age/ Sex | Subtype | CGH results[a] |
|---|---|---|---|
| 1 | 64/F | Adenoma | +4,+5,+X,+7,+9,+12,+14q12–qter, +16,+17,+22q12.1–qter |
| 2 | 77/F | Adenoma | Normal |
| 3 | 59/F | Adenoma (oxyphilic) | +5p14–pter,+5q23.1–qter,+7 |
| 4 | 35/F | Adenoma | +5,+X,+7,+9,+12,+14q12–qter,+16, +17,+18,+20,+21q21.3–qter |
| 5 | 56/F | Adenoma (oxyphilic) | +5,+7,+10,+13,+14,+17,+18 |
| 6 | 34/F | Adenoma | Normal |
| 7 | 37/F | Adenoma (oxyphilic) | Normal |
| 8 | 58/F | Adenoma | +3,+5,+X,+7,+12,+18 |
| 9 | 47/F | Adenoma | Normal |
| 10 | 43/F | Adenoma | +5p14–pter,+5q23–qter,+7,+12 |
| 11 | 25/F | Adenoma | +X,+7,+9,+12,+14q13–qter,+17 |
| 12 | 47/F | Adenoma | +5,+7,+12,+14q21–qter |
| 13 | 47/F | Adenoma | –3 |
| 14 | 49/F | Adenoma | Normal |
| 15 | 36/F | Adenoma | Normal |
| 16 | 41/F | Adenoma (oxyphilic) | +7 |
| 17 | 43/M | Adenoma | Normal |
| 18 | 76/F | Adenoma | Normal |
| 19 | 54/M | Adenoma | Normal |
| 20 | 41/M | Adenoma | Normal |
| 21 | 46/F | Adenoma | +3,+5,+X,+7,+12,+13,+14, +16,+17,+18,+19,+20 |
| 22 | 43/F | Adenoma | –22 |
| 23 | 44/F | Adenoma | Normal |
| 24 | 73/F | Adenoma | Normal |
| 25 | 26/F | Adenoma (atypical) | –22 |
| 26 | 39/M | Adenoma (atypical) | –1,–2,–6,–9,–11,–13 |
| 27 | 46/F | Adenoma (atypical) | Normal |
| 28 | 48/F | Adenoma (atypical) | Normal |
| 29 | 87/F | Adenoma (atypical) | Normal |
| 30 | 34/F | Carcinoma (minimally invasive) | Normal |
| 31 | 78/F | Carcinoma (minimally invasive) | Normal |
| 32 | 66/F | Carcinoma (minimally invasive) | +17q, –22q12.3–qter |
| 33 | 53/M | Carcinoma (minimally invasive) | +17q |
| 34 | 57/F | Carcinoma (minimally invasive) | Normal |
| 35 | 50/M | Carcinoma (widely invasive) | +1q22–qter,–1p21–22 |
| 36 | 76/M | Carcinoma (widely invasive) | –13,–22 |
| 37 | 77/F | Carcinoma (widely invasive) | –22 |
| 38 | 81/F | Carcinoma (widely invasive) | +1q22–qter,–1p,–22 |
| 39 | 71/F | Carcinoma (widely invasive) | Normal |
| 40 | 79/F | Carcinoma (widely invasive) | –22 |
| 41 | 85/F | Carcinoma (widely invasive) | –1p13–23,–21,–22 |
| 42 | 68/F | Carcinoma (widely invasive) | +1q24–qter,–1p,–9,–13,–18,–22 |

[a]Gains of the DNA sequences are marked with + and losses with –.

© Cancer Research Campaign 1998

**Figure 1** Mean red–green ratio profiles of selected chromosomes reflecting DNA sequence copy number changes in follicular adenoma and follicular carcinoma. Profiles are those of chromosomes 5, 7, 12, 14 (follicular adenoma) and 1, 22 (follicular carcinoma), which showed the most frequent genetic changes. The line in the middle of the profile indicates the base line ratio (1.0), the left and the right lines indicate ratio values of 0.85 and 1.17 respectively. Left: The profiles represent the following aberrations: loss of 1p and gain of 1q (case no. 42), gain of entire chromosome 5 (case no. 8), gain of entire chromosome 7 (case no. 21), gain of entire chromosome 12 (case no. 8), gain of 14q12–qter (case no. 1) and loss of entire chromosome 22 (case no. 42). Right: The profiles of chromosomes with no aberrations obtained from various negative control experiments from the analysis (e.g. chromosomes that were heavily bent or overlapping or those that had overlying artefacts). Chromosomal regions were interpreted as amplified (a gain) when the red–green ratio exceeded 1.17; as highly amplified, when the ratio exceeded 1.5; and underrepresented (a loss) when the ratio was less than 0.85 (Figure 1). All findings were confirmed using a confidence interval of 99%. A positive control with known chromosomal aberrations and a negative control were included in each hybridization to verify the reliability of the method. Chromosomal regions in the centromeric areas of chromosomes 1, 9, 16 and Y and the p-arms of acrocentric chromosomes were discarded from the analysis because of their large heterochromatic areas.

As CGH recognizes only proportional changes in DNA copy number, the ratio profiles do not indicate the absolute copy number changes. In diploid and near-diploid cells, a ratio of 1.5 indicates a 100% increase in the copy number in a chromosome arm or in an area of the size of a chromosome band (Knuutila et al, 1998). If this threshold is not reached the increase is only 50%, suggesting chromosomal trisomy.

### Statistical analysis

Fisher's exact test was used in statistical analysis. All *P*-values are two-tailed.

### RESULTS

A summary of all gains and losses detected is shown in Table 1 and Figure 2. As many as 14 (48%) of the 29 follicular adenomas had

© Cancer Research Campaign 1998

DNA copy number changes. Only nine (13%) of the total of 72 changes found in adenomas were losses, and they occurred only in four (14%) adenomas, whereas gains were found in ten (35%) adenomas. On average, there were 2.5 changes per adenoma (median 1; range 0–12). A gain in DNA sequences occurred in 16 different chromosomes, and a gain of a whole chromosome was very common (74% of all changes), probably related to trisomy of chromosomes 3, 4, 5, 7, 9, 10, 12, 13, 14, 16, 17, 18, 19, 20 and X.

The most frequently involved chromosomes in follicular adenoma were 7 (ten cases; 34%) and 5 (eight cases; 28%). Particularly, a gain of the entire chromosome 7 was present in all ten adenomas that displayed one or more gains. The other chromosomes commonly gained were 12 (seven cases; 24%), 14 (six cases; 21%), X (five cases; 17%), 17 (five cases; 17%), 18 (four cases; 14%) and 9 (three cases; 10%). In four cases, the only changes were a loss of an entire chromosome or chromosomes. These losses were in chromosomes 1, 2, 6, 11, 13 (case 26), 3 (case 13) and 22 (cases 22 and 25). Two of these four follicular adenomas with a loss (nos. 25 and 26) were histologically classified as atypical adenomas. A DNA copy number gain was not found in any of the five atypical follicular adenomas.

Follicular carcinomas displayed copy number changes in 9 (69%) out of the 13 cases analysed. On average, there were 1.6 changes per case (median 1; range 0–6), and the number of changes detected among the 13 cases was 21. Unlike adenomas, losses were more common than gains (16 vs. 5), and the frequency of tumours with a loss (8 out of 13, 62%) was greater than in adenomas (4 out of 29, 14%, $P = 0.003$). Aberrations were detected in eight different chromosomes: 1, 9, 13, 17, 18, 19, 21 and 22. Chromosome 22 was most frequently involved, and it was deleted in as many as six (46%) carcinomas. Five of these six tumours showed a loss of the whole chromosome 22, and in one case there was a loss of the chromosome arm 22q12.3–qter. Loss of chromosome 22 or 22q was present in six of the eight widely invasive follicular carcinomas, but only in one of the five minimally invasive carcinomas. In adenomas, a loss of chromosome 22 was found only in two (7%) tumours ($P = 0.002$), one of which was an atypical adenoma. A loss of 1p, 1p21–22 and 1p13–23 was detected in four carcinomas (cases 35, 38, 41 and 42). In addition, loss of the whole chromosome 9, 18 or 19 were each detected in one carcinoma, and chromosome 13 in two carcinomas. A gain was found only in four chromosomes, 1q22–qter (cases 35, 38), 1q24–qter (case 42) and the long arm of chromosome 17 (cases 32, 33).

**Figure 2** A summary of gains and losses in follicular adenomas and carcinomas. The gains are shown on the right side of the chromosomes, and the losses on the left. Each line represents a gained or lost region in a single tumour. The gains and losses found in the follicular adenomas are marked with solid lines, and those in follicular carcinomas with dotted lines

© Cancer Research Campaign 1998

No highly amplified chromosomal regions were detected either in follicular adenoma or carcinoma. None of the carcinomas had aberrations in chromosome 7, which was gained in 10 out of the 29 adenomas ($P = 0.02$), and no gains were found in chromosomes 5, 12, 14 or X, which were also frequently gained in adenomas. There were only four losses that were found both in adenomas and carcinomas. They were found in chromosomes 1 (one adenoma and four carcinomas), 9 (one adenoma and one carcinoma), 13 (one adenoma and two carcinomas) and 22 (two adenomas and seven carcinomas). The only gain that was found in both types of tumours was that of chromosome 17q (five adenomas and two carcinomas).

### DISCUSSION

We found DNA copy number changes to be frequent in follicular thyroid adenomas. Typical aberrations were gains of the entire chromosomes 5, X, 7, 12, 14, 17 and 18. Our results are in line with results of karyotype studies in which numerical chromosomal abnormalities, mainly trisomies, and especially trisomies of chromosomes 5, 7 and 12, have been described (Antonini et al, 1993; Belge et al, 1994; Heim et al, 1995). In a fluorescence in situ hybridization study, not only one extra copy but also several extra copies of chromosomes 7 and 12 were reported (Criado et al, 1995). Our CGH data did not, however, suggest several extra copies in these chromosomes, as high DNA copy number changes were not detected (see Materials and methods, section Digital image analysis).

In follicular carcinoma, DNA copy number losses were commonly found. A typical aberration was deletion of a part of or the entire chromosome 22, which was found in about one-half of all carcinomas. It appeared to be more frequent in widely invasive than in minimally invasive carcinomas, which suggests that this deletion may be associated with malignant progression of follicular carcinoma. Further studies are needed to find out if deletion of chromosome 22 is correlated to survival.

Also in karyotype studies, deletions have been found in follicular carcinomas (Jenkins et al, 1990). A monosomy or DNA copy number loss of chromosome 22 is not unique to follicular thyroid carcinomas as it has been described in other types of human neoplasms such as meningioma, glioma, mesothelioma and gastrointestinal stromal tumour (Tonk et al, 1992; Mohapatra et al, 1995; El-Rifai et al, 1996; Björqkvist et al, 1997). The long arm of chromosome 22 contains the tumour suppressor gene neurofibromatosis type 2 (NF2) at 22q12 (Ruttledge et al, 1994), but there is also evidence for the presence of another putative tumour suppressor gene distal to NF2 (Schofield et al, 1996). The significance of these and other suppressor genes located in 22q in the genesis of follicular thyroid carcinoma is unsettled.

Although the CGH profiles of most follicular adenomas and carcinomas differed greatly from each other, in four follicular adenomas the only aberration detected was a loss of an entire chromosome or chromosomes, and in two of them the lost chromosome was 22. Of the five atypical adenomas, losses were found in two, and in one of them chromosome 22 had been lost. These findings might suggest that some follicular adenomas, including the atypical adenoma, may have a common genetic origin with follicular carcinoma. Atypical adenoma is not a well-defined entity, but rather a tumour that shows architectural and cytological features resembling those seen in follicular carcinoma. It, however, lacks invasion, the most important criterion of follicular carcinoma. In earlier studies, a subgroup of follicular carcinoma called follicular carcinoma without invasion was described (Woolner et al, 1961). These tumours were associated with excellent prognosis and would probably be called atypical adenomas at present.

In conclusion, the results indicate that in follicular thyroid adenomas extensive chromosomal changes are often present, and that these changes are mainly gains of entire chromosomes. Unlike adenomas, gains are not frequent in follicular carcinoma, whereas losses, especially those of chromosome 22 or 22q, are found often. Loss of chromosome 22 may be associated with the widely invasive type of follicular carcinoma.

### ACKNOWLEDGEMENTS

The skilful assistance of Päivi Heino and Elina Roimaa is gratefully acknowledged. We also thank Anna-Maria Björqkvist. The study was supported by the Finnish Cancer Society (SH, V-MW), Helsinki University Central Hospital Research Fund (KF, SH) and the Emil Aaltonen Foundation (SH).

### REFERENCES

Antonini P, Lévy N, Caillou B, Vénuat A-M, Schlumberger M, Parmentier C and Bernheim A (1993) Numerical aberrations, including trisomy 22 as the sole anomaly, are recurrent in follicular thyroid adenomas. *Genes Chrom Cancer* **8**: 63–66

Belge G, Thode B, Rippe V, Bartnizke S and Bullerdiek J (1994) A characteristic sequence of trisomies starting with trisomy 7 in benign thyroid tumours. *Hum Genet* **94**: 198–202

Björqkvist A-M, Tammilehto L, Anttila S, Mattson K and Knuutila S (1997) Recurrent DNA copy number changes in 1q, 4q, 6q, 9q, 13q, 14q and 22q detected by comparative genomic hybridization in malignant mesothelioma. *Br J Cancer* **75**: 523–527

Bondeson L, Bengtsson A, Bondeson A-G, Dahlenfors R, Grimelius L, Wedell B and Mark J (1989) Chromosome studies in thyroid neoplasia. *Cancer* **64**: 680–685

Oriado B, Barros A, Suijkerbuijk RF, Weghuis DO, Seruca R, Fonseca E and Castedo S (1995) Detection of numerical alterations for chromosomes 7 and 12 in benign thyroid lesions by in situ hybridization. *Am J Pathol* **147**: 136–144

El-Rifai W, Sarlomo-Rikala M, Miettinen M, Knuutila S and Andersson L (1996) DNA copy number losses in chromosome 14: an early change in gastrointestinal stromal tumours. *Cancer Res* **56**: 3230–3233

El-Rifai W, Larramendy ML, Björkqvist A-M, Hemmer S and Knuutila S (1997) Optimization of comparative genomic hybridization by fluorochrome conjugated to dCTP and dUTP nucleotides. *Lab Invest* **77**: 699–700

Franssila K (1997) Thyroid. In *Bloodworth's Endocrine Pathology*, 3rd edn, Lechago J and Gould VE (eds), pp. 171–247. Williams & Wilkins: Baltimore

Grebe SKG, Mciver B, Hay ID, Wu PSC, Maciel LMZ, Drabkin HA, Goellner JR, Grant CS, Jenkins RB and Eberhardt NL (1997) Frequent loss of heterozygosity on chromosomes 3p and 17p without VHL or p53 mutations suggests involvement of unidentified tumor suppressor genes in follicular thyroid carcinoma. *J Clin Endocrinol Metab* **82**: 3684–3691

Hedinger CE, Williams ED and Sobin LH (1988) *Histological Typing of Thyroid Tumours*. Springer Verlag: New York

Hedinger C, Williams ED and Sobin LH (1989) The WHO histological classfication of thyroid tumours: a commentary on the second edition. *Cancer* **63**: 908–911

Heim S and Mitelman F (1995) *Cancer Cytogenet* 2nd edn. Wiley-Liss: New York

Herrman MA, Hay ID, Bartelt Jr DH, Ritland SR, Dahl RJ, Grant CS and Jenkins RB (1991) Cytogenetic and molecular genetic studies of follicular and papillary thyroid cancers. *J Clin Invest* **88**: 1596–1604

Jenkins RB, Hay ID, Herath JF, Schultz CG, Spurbeck JL, Grant CS, Goellner JR and Dewald GW (1990) Frequent occurrence of cytogenetic abnormalities in sporadic non-medullary thyroid carcinoma. *Cancer* **66**: 1213–1220

Joensuu H and Klemi PJ (1988) DNA aneuploidy in adenomas of endocrine organs. *Am J Pathol* **132**: 145–151

Kallioniemi O-P, Kallioniemi A, Piper J, Isola J, Waldman FM, Gray JW and Pinkel D (1994) Optimizing comparative genomic hybridization for analysis of DNA sequence copy number changes in solid tumour. *Genes Chrom Cancer* **10**: 231–243

© Cancer Research Campaign 1998

Knuutila S. Björkqvist A-M. Autio K. Tarkkanen M. Wolf M. Monni O. Szymanska J. Larramendy ML. Tapper J. Pere H. El-Rifai W. Hemmer S. Wasenius V-M. Vidgren V and Zhu Y (1998) DNA copy number amplifications in human neoplasms – a review of comparative genomic hybridization studies. *Am J Pathol* **152**: 1107–1123

Mohapatra G. Kim DH and Feurstein BG (1995) Detection of multiple gains and losses of genetic material in ten glioma cell lines by comparative genomic hybridization. *Genes Chrom Cancer* **13**: 86–93

Roque L. Castedo S. Gomes P. Soares P. Clode A and Soares J (1993*a*) Cytogenetic findings in 18 follicular thyroid adenomas. *Cancer Genet Cytogenet* **67**: 1–6

Roque L. Gomes P. Correia C. Soares P. Soares J and Castedo S (1993*b*) Thyroid nodular hyperplasia: chromosomal studies in 14 cases. *Cancer Genet Cytogenet* **69**: 31–34

Roque L. Castedo S. Clode A and Soares J (1993*c*) Deletion of 3p25–pter in a primary follicular thyroid carcinoma and its metastasis. *Genes Chrom Cancer* **8**: 199–203

Ruttledge M. Sarrazin J. Rangaratnam S. Phelan C. Twist E. Merel P. Delattre O. Thomas G. Nordenskjöld M. Collins P. Dumanski J and Rouleau G (1994) Evidence for the complete inactivation of the NF2 gene in the majority of sporadic meningiomas. *Nature Genet* **6**: 180–184

Sambrook J. Fritisch EF and Maniatis T (1989) *Molecular Cloning: a Laboratory Manual*. 2nd edn. Cold Spring Harbor Labarotory Press: Cold Spring Harbor. NY

Schofield DE. Beckwith JB and Sklar J (1996) Loss of heterozygosity at chromosome regions 22q11–12 and 11p15.5 in renal rhabdoid tumours. *Genes Chrom Cancer* **15**: 10–17

Sozzi G. Miozzo M. Cariani TC. Bongarzone I. Pilotti S. Pierotti MA and Della Porta G (1992) A t(2:3)(q12–13:p24–25) in follicular thyroid adenomas. *Cancer Genet Cytogenet* **64**: 38–41

Teyssier J-R. Liautaud-Roger F. Ferre D. Patey M and Dufer J (1990) Chromosomal changes in thyroid tumours. Relation with DNA content. karyotypic features. and clinical data. *Cancer Genet Cytogenet* **50**: 249–263

Tonk V. Osella P. Delasmorenas A. Wyandt HE and Milunsky A (1992) Abnormalities of chromosome 22 in meningiomas and confirmation of the origin of a dicentric 22 by in situ hybridization. *Cancer Genet Cytogenet* **64**: 65–68

van den Berg E. Oosterhuis JW. de Jong B. Buist J. Vos AM. Dam A and Vermeij B (1990) Cytogenetics of thyroid follicular adenomas. *Cancer Genet Cytogenet* **44**: 217–222

van den Berg E. van Doormaal JJ. Oosterhuis JW. de Jong B. Wiersema J. Vos AM. Dam A and Vermeij A (1991) Chromosomal aberrations in follicular thyroid carcinoma. Case report of a primary tumor and its metastasis. *Cancer Genet Cytogenet* **54**: 215–222

Woolner LB. Beahrs OH. Black BM. McConahey WM and Keating Jr FR (1961) Classification and prognosis of thyroid carcinoma. *Am J Surg* **102**: 354–387

© Cancer Research Campaign 1998